# Analysis of the impact of rotation error on PTV margins in multiple brain metastases fractionated stereotactic radiotherapy based on single-isocenter multi-target technique

**DOI:** 10.3389/fonc.2025.1564126

**Published:** 2025-04-25

**Authors:** Yuhong Li, Rui Hua, Liling Dai, Wei Chen, Junyuan Zhang, Qian Wang, Yufeng Xu, Tingting Wang

**Affiliations:** ^1^ Department of Radiation Oncology, Affiliated Cancer Hospital of Nanjing Medical University, Jiangsu Cancer Hospital, Jiangsu Institute of Cancer Research, Nanjing, Jiangsu, China; ^2^ Department of Science and Technology, Affiliated Cancer Hospital of Nanjing Medical University, Jiangsu Cancer Hospital, Jiangsu Institute of Cancer Research, Nanjing, Jiangsu, China; ^3^ Department of Thoracic Surgery, Affiliated Cancer Hospital of Nanjing Medical University, Jiangsu Cancer Hospital, Jiangsu Institute of Cancer Research, Nanjing, Jiangsu, China

**Keywords:** single-isocenter multi-target, fractionated stereotactic radiotherapy, volumetric modulated arc therapy, multiple brain metastases, rotational errors, planning target volume margin expansion

## Abstract

**Background:**

Rotational error cannot be overlooked in single-isocenter multi-target (SIMT) stereotactic radiotherapy. This retrospective study aimed to evaluate the treatment accuracy of linear accelerator-based fractionated stereotactic radiotherapy (FSRT) using SIMT non-coplanar volumetric modulated arc therapy (VMAT) in patients with multiple brain metastases. We explored the impact of rotational error on planning target volume (PTV) margins, providing clinical evidence for the selection of appropriate PTV margin values.

**Methods:**

A total of 161 patients with multiple brain metastases (733 treatments; actual clinical PTV margins ranged from 1~2 mm) were included. Theoretical PTV margins were calculated based on the Van Herk and Jenghwa Chang formulas. We analyzed the influence of the distance from each target to the treatment isocenter, rotational errors, and PTV margin on treatment outcomes. Additionally, individualized PTV margins for each patient were calculated using the Jenghwa Chang formula and patients were divided into subgroups according to a 2-mm threshold for further analysis.

**Results:**

The mean residual translational setup errors ranged from –0.04~0.01 mm, and rotational setup errors ranged from 0.15°~0.49°, both within acceptable limits. According to the Van Herk formula, required margins in posterior-anterior, superior-inferior, and right-left directions were 1.44 mm, 1.68 mm, and 1.78 mm, respectively. By incorporating both translational and rotational errors using the Jenghwa Chang formula, the comprehensive margin ranged from 1.69~1.79 mm (calculated based on the 95% confidence interval of distances from targets to isocenter). Additionally, when the mean distance from all targets to their respective treatment isocenters was 30.62 mm, the required margin calculated solely for translational errors using the Jenghwa Chang formula was 1.23 mm; if rotational errors were neglected, target coverage probability would decrease from 95% to 73%. Further subgroup analysis showed that 25 patients whose individualized theoretical margins exceeded 2 mm tended to experience worse outcomes compared to others, including intracranial local failure (ILF, defined as lesion progression within the previously irradiated intracranial region during follow-up; 32.00% vs. 22.29%, *P = 0.32*), one-year local control (64.00% vs. 65.44%, *P = 0.89*), and one-year intracranial progression-free survival (iPFS, 44.00% vs. 51.45%, *P = 0.85*). However, these differences did not reach statistical significance.

**Conclusion:**

This study confirms that the SIMT non-coplanar VMAT technique ensures treatment accuracy for FSRT in multiple brain metastases. Rotational errors reduce dose coverage, and a minimum safety margin of 1.79 mm is recommended to ensure tumor coverage and reduce local failure, providing a basis for future treatment optimization.

## 1 Introduction

It is estimated that approximately 30% of cancer patients will develop brain metastases during the course of their disease, and with the optimization of cancer treatment methods in recent years, the incidence of brain metastases continues to rise (1). Among these, more than half of the brain metastases are multiple lesions (the number of lesions ≥ 2) (2). For multiple lesions, especially when the cumulative volume of the metastases is large, an increasing number of studies suggest using fractionated stereotactic radiotherapy (FSRT) for treatment (3, 4). FSRT is characterized by a single high-dose fraction and a steep dose gradient at the tumor boundary, making precise localization during treatment particularly crucial. Even small positioning errors can alter the overall dose distribution in the target area, significantly reducing the conformity of the dose to the target and thereby affecting treatment outcomes (5). Although FSRT based on linear accelerators, combined with image-guided technologies such as KV cone-beam computed tomography (KV-CBCT) and six-degree-of-freedom (6-DOF) treatment couch, can effectively correct initial setup errors to the maximum extent possible, residual setup errors may still result in decreased target dose coverage, which in turn increases the risk of tumor recurrence (6–9).

In the past, FSRT for multiple brain metastases typically employed a multi-isocenter approach, where each target was treated individually, resulting in treatment durations of several hours per session. To improve treatment efficiency, the single-isocenter multiple-target (SIMT) non-coplanar volumetric modulated arc therapy (VMAT) technique has been increasingly applied in FSRT for multiple brain metastases. Unlike the multi-isocenter approach, the SIMT technique uses a single isocenter located at the geometric center of the total planning target volume (PTV), allowing for the treatment of multiple targets simultaneously. This approach not only reduces patient imaging doses but also achieves similar dosimetric effects to the former method (10, 11). However, the implementation of SIMT technology may introduce additional rotational errors that affect the dose distribution. Correcting these errors across all target areas simultaneously using KV-CBCT and a 6-DOF treatment couch is challenging (6–8). The International Commission on Radiation Units and Measurements Report 50 recommends increasing the expansion margin to ensure adequate dose coverage for the tumor target area, thus generating the PTV. Traditionally, the formula proposed by Van Herk et al. has been widely used for quantitatively analyzing the expansion margin required for the gross tumor volume (GTV) or clinical target volume (12). Additionally, some studies have suggested increasing the expansion margin by 0~3 mm on the basis of GTV to accommodate setup errors (13). However, given the technical differences across various radiation therapy centers, and the fact that the Van Herk formula does not account for the adverse effects of rotational errors in SIMT techniques, there is currently no consensus in the field regarding the exact definition of the expansion margin. In recent years, several research teams have explored the expansion margin from GTV to PTV (14–18). Jenghwa Chang, using a statistical modeling approach, proposed a new formula for calculating the expansion margin, which takes both translational and rotational errors into account. The resulting value ensures that the probability of GTV being included within the corresponding PTV region is greater than the pre-set coverage probability (15, 16, 19).

Based on the aforementioned background, this study aims to retrospectively analyze the application of FSRT for multiple brain metastases in our center, utilizing a linear accelerator and SIMT non-coplanar VMAT technology, and to assess its treatment accuracy. Differences in PTV margins calculated by the Van Herk and Jenghwa Chang formulas were compared, and the impacts of distances from targets to the treatment isocenter, rotational errors, and PTV margins on patient outcomes were analyzed to provide guidance for selecting clinical PTV margins in our center.

## 2 Materials and methods

### 2.1 Patient cohort and treatment characteristics

This study retrospectively analyzed patients with multiple brain metastases who underwent FSRT at Jiangsu Cancer Hospital from January 2021 to December 2023. The study was approved by the Ethics Committee of Jiangsu Cancer Hospital. Inclusion criteria were as follows: (1) FSRT was performed on all patients using the Varian TrueBeam linear accelerator equipped with the HyperArc system; (2) non-coplanar radiation fields and SIMT-VMAT were used; (3) patients had more than one brain metastasis; (4) patients were able to undergo follow-up every 2~3 months via telephone, outpatient visits, or hospitalization. Exclusion criteria included: (1) brainstem or leptomeningeal metastases; (2) primary pathology of small cell lung cancer; (3) treatment interruption; (4) failure to adhere to KV-CBCT imaging verification and 6-DOF treatment couch correction protocols.

All patients were immobilized in a supine position using a No.1 carbon fiber baseplate (model: Q-Fix Pro-Lok, dose attenuation rate <0.5%) combined with an Orfit integrated head fixation system. The fixation system included a thermoplastic head mask anchored at three points (forehead and bilateral temporal regions; thickness: 3.5 mm; repositioning accuracy ≤ 0.5 mm) and an adjustable-angle S-shaped headrest (sagittal deviation ≤ 1.0 mm; tilt angle range: –10° to +30°). After initial positioning, the treatment isocenter was marked on the mask using a laser positioning system to ensure daily reproducibility. Contrast-enhanced computed tomography (CT) (range: from cranial vertex to C4 vertebra, 512 × 512 matrix, slice thickness: 1 mm, spiral pitch: 0.8) and 3.0T magnetic resonance imaging with T1-weighted contrast-enhanced sequences (TR/TE = 500/15 ms, slice thickness: 1 mm, FOV: 240 × 240 mm) were fused using a mutual-information rigid registration algorithm (mean squared error < 0.8 mm), verified independently by two radiation oncologists. The GTV was delineated on fused images, defined as clearly identifiable tumor areas (lesions unclear on planning images were excluded). The PTV was created by expanding the GTV by 1~2 mm. A multileaf collimator with leaf width of 2.5 mm (dynamic positioning accuracy ≤ 0.5 mm verified daily by electronic portal imaging device) and maximum collimator diameter of 1.7 cm was employed. Cone-beam computed tomography (CBCT)-guided setup verification with 6-DOF corrections (bone and soft-tissue dual-mode registration) was conducted before each treatment; residual errors were required to be ≤ 2 mm (translation) and ≤ 3° (rotation). An optical surface monitoring system tracked forehead landmarks in real-time during treatment, automatically pausing irradiation upon displacement exceeding 1.0 mm. Treatment utilized 6 MeV-X rays in flattening filter-free mode with a maximum dose rate of 2400 MU/min. Dosimetric verification employed an ArcCHECK phantom (passing criteria: ≥ 95% for 3%/2 mm) and ion-chamber point-dose measurements (deviation ≤ ± 2%). The treatment plan required that over 95% of the PTV area receive the prescribed dose, with the dose distribution approximating a Gaussian curve. The low dose outside the PTV should be uniformly distributed around the target, and the dose fall-off should be controlled at greater than 10% per 3 mm. Additionally, the dose in the region outside the PTV should not exceed the prescribed dose. The 10 Gy isodose lines of two adjacent target volumes should not overlap. Dose limits for critical organs followed the RTOG9005 guidelines (20).

### 2.2 Data collection and processing

All treatment plans were generated using the Eclipse planning system with the Acuros dose algorithm. With the treatment isocenter as the origin of a three-dimensional coordinate system, the coordinates  (x,y,z) of the geometric center of each patient’s targets were recorded, and distances (d) from the isocenter to each target were calculated using the Euclidean formula (Equation 1). Subsequently, the mean distance of all targets to the isocenter for each patient ( $\bar{d}$ ) was calculated using Equation 2. Additionally, the overall mean distance (D) for all patients was obtained using Equation 3. Residual setup errors in six directions were recorded for each treatment: rotational errors ${\text{R}}_{\text{Roll}}$ , ${\text{R}}_{\text{Pitch}}$ , and ${\text{R}}_{\text{Yaw}}$ correspond to roll, pitch, and yaw directions, respectively; translational errors ${\text{T}}_{\text{IS}}$ , $\;{\text{T}}_{\text{RL}}$ and ${\text{T}}_{\text{PA}}$ correspond to superior–inferior, right–left, and posterior–anterior directions, respectively. The total translational error ( ${\text{T}}_{\text{total}}$ , in mm) for each treatment session was calculated using Equation 4, for subsequent error analysis.


(1) $$\begin{array}{c} d=\sqrt{x^{2}+y^{2}+z^{2}} \end{array}$$

(2) $$\bar{d}=\frac{1}{n}\sum_{i=1}^{n}\sqrt{x_{i}^{2}+y_{i}^{2}+z_{i}^{2}}$$

In Equation 2, $\left({\text{x}}_{\text{i}},{\text{y}}_{\text{i}},{\text{z}}_{\text{i}}\right)$ represents the three-dimensional coordinates of the geometric center of the i-th brain metastasis relative to the treatment isocenter for each patient, and n represents the number of brain metastases for that patient.


(3) $$D=\frac{1}{N}\sum_{j=1}^{N}d_{j}$$

In Equation 3, ${\text{d}}_{\text{j}}$ denotes the distance between the geometric center of the j-th brain metastasis and its respective treatment isocenter, and N denotes the total number of lesions across all patients enrolled in the study.


(4) $$\begin{array}{c} T_{total}=\sqrt{T_{IS}^{2}+T_{RL}^{2}+T_{PA}^{2}} \end{array}$$

In addition, based on the error angles of the patient in the three rotational directions ( ${\text{R}}_{\text{Roll}}$ , $\;{\text{R}}_{\text{Pitch}}$ , $\;{\text{R}}_{\text{Yaw}}$ ) during each treatment, the corresponding rotation matrix ℛ was constructed:


(5) $$\begin{array}{c} \text{R}={\text{ℛ}}_{\text{x}}\left(R_{Roll}\right){\text{ℛ}}_{\text{y}}\left(R_{Pitch}\right){\text{ℛ}}_{\text{z}}\left(R_{Yaw}\right) \end{array}$$

Where ${\text{ℛ}}_{\text{x}}\left({\text{R}}_{\text{Roll}}\right)$ , ${\text{ℛ}}_{y}\left({\text{R}}_{\text{Pitch}}\right)$ , ${\text{ℛ}}_{\text{y}}\left({\text{R}}_{\text{Yaw}}\right)$ represent the rotation matrices for the rotation angles ${\text{R}}_{\text{Roll}},{\text{ R}}_{\text{Pitch}}\;$ and ${\text{R}}_{\text{Yaw}}$ (in °) around the x-axis, y-axis, and z-axis, respectively. Subsequently, the rotation matrix ℛ was converted into a rotation vector V using Rodrigues’ rotation formula Equation 6. The direction of V denotes the rotation axis, represented by the unit vector U Equation 7, and its magnitude corresponds to the total rotation angle ${\text{R}}_{\text{total}}$ (in °):


(6) $$\begin{array}{c} 𝒱=\frac{1}{2\sin(R_{total})}\left[\begin{array}{ccc} {ℛ}_{32} & - & {ℛ}_{23}\\ {ℛ}_{13} & - & {ℛ}_{31}\\ {ℛ}_{21} & - & {ℛ}_{12} \end{array}\right] \end{array}$$

(7) $$\begin{array}{c} 𝒱=R_{totol}𝒰 \end{array}$$

(8) $$\begin{array}{c} R_{total}=\cos^{-1}(\frac{trace\left(ℛ\right)-1}{2}) \end{array}$$

Where trace(ℛ) is the trace of the rotation matrix ℛ , and ${ℛ}_{\text{ij}}$ is the element at the i-th row and j-th column of the rotation matrix ℛ .

Using Equations 5 **~** 8, the rotational errors from each treatment fraction were integrated into a single comprehensive rotational angle ( ${\text{R}}_{\text{total}}$ ) for subsequent analysis of the PTV margin.

### 2.3 Calculation of PTV margin expansion

According to Van Herk et al., in order to ensure that at least 90% of patients’ PTVs receive 95% of the prescribed dose, the margin expansion is calculated as follows (12):


(9) $$\begin{array}{c} M_{Van\;Herk}=2.5\Sigma +0.7\sigma \end{array}$$

where ${\text{M}}_{\text{Van Herk}}$ represents the calculated margin, Σ (in mm) is the population systematic error, and σ (in mm) is the population random error. Given the limited fractions per patient and relatively small inter-individual variations, the group mean of the random error standard deviation (SD) is typically used to compute σ (Equation 11). The calculations of Σ and σ are as follows:


(10) $$\sum =\sqrt{\frac{P}{N(P-1)}\sum_{p=1}^{P}F_{p}{\left(m_{p}-\bar{m}\right)}^{2}}$$

(11) $$\begin{array}{c} \begin{array}{c} \sigma =\sqrt{\frac{1}{N-P}\sum_{p=1}^{P}\left(F_{p}-1\right)\cdot \sigma_{p}^{2}}\\ =\sqrt{\frac{1}{N-P}\sum_{p=1}^{P}\sum_{f=1}^{Fp}{\left(x_{pf}-m_{p}\right)}^{2}} \end{array} \end{array}$$

where N is the total number of fractions, P is the total number of patients, ${\text{F}}_{\text{p}}$ is the number of fractions for patient p, ${\text{x}}_{\text{pf}}$ (in mm) is the translational error along the x-axis for patient p in fraction f, ${\text{σ}}_{\text{p}}$ (in mm) is the SD of random errors for patient p, ${\text{m}}_{\text{p}}$ (in mm) is the mean translational error (systematic error) for patient p, and $\bar{m}$ (in mm) is the mean systematic error across all patients.

Jenghwa Chang’s method accounts for rotational uncertainties, assuming random errors in translation and rotation that follow independent three-dimensional normal distributions (15, 16, 19). Using Chang’s approach, the comprehensive PTV margin considering both translational and rotational uncertainties ( ${\text{M}}_{\text{trans}+\text{rot}}$ ) was calculated for all 161 patients as follows:


(12) $$\begin{array}{c} \begin{array}{c} M_{trans+rot}=\sqrt{M_{trans}^{2}+M_{rot}^{2}}\\ =\sqrt{{\left(\chi_{\alpha }\cdot \sigma_{T_{total}}\right)}^{2}+{\left(\chi_{\alpha }\cdot \sigma_{r_{total}}\right)}^{2}}\\ =\chi_{\alpha }\sqrt{\sigma_{T_{total}}^{2}+\sigma_{r_{total}}^{2}}\\ =\chi_{\alpha }\sqrt{\sigma_{T_{total}}^{2}+{\left(0.01424\cdot \bar{d}\cdot \sigma_{R_{total}}\right)}^{2}} \end{array} \end{array}$$

Where ${\text{M}}_{\text{trans}}$ and ${\text{M}}_{\text{rot}}$ represent the PTV margin expansions required to accommodate translational and rotational errors, respectively Equation 12. ${\text{χ}}_{\text{α}}$ represents the value corresponding to the probability that the GTV is covered by the PTV. When the probability of the GTV being within the PTV is at least 95%, ${\text{χ}}_{\text{α}}$ is set to 2.795. ${\text{σ}}_{{\text{T}}_{\text{total}}}$ is the standard deviation of the total translational error ( ${\text{T}}_{\text{total}}$ ) for all 733 treatments across all patients (The calculation of ${\text{T}}_{\text{total}}$ is shown in Equation 4). ${\text{σ}}_{{\text{r}}_{\text{total}}}$ (in mm) represents the rotational uncertainty for all 733 treatments, and ${\text{σ}}_{{\text{R}}_{\text{total}}}$ (in °) represents the standard deviation of the total rotation error ( ${\text{R}}_{\text{total}}$ ) for all 733 treatments.

### 2.4 Grouping and endpoints

Considering individual differences, the study calculated individualized PTV margin expansion for each patient using the Jenghwa Chang formula, denoted as ${\text{M}}_{\text{trans}+\text{rot}}^{'}$ , with the following calculation process:


(13) $$\begin{array}{c} \begin{array}{c} M_{trans+rot}^{'}=\chi_{\alpha }\sqrt{{\left(\sigma_{T_{total}}^{'}\right)}^{2}+{\left(0.01424\cdot \bar{d}\cdot \sigma_{R_{total}}^{'}\right)}^{2}} \end{array} \end{array}$$

Where ${\text{σ}}_{{\text{T}}_{\text{total}}}^{'}$ represents the standard deviation of the total translational error ( ${\text{T}}_{\text{total}}$ ) across all treatment fractions for each patient. ${\text{σ}}_{{\text{R}}_{\text{total}}}^{'}$ (in °) represents the standard deviation of the total rotation error ( ${\text{R}}_{\text{total}}$ ) across all treatment fractions for each patient.

Actual margins applied in this study ranged from 1~2 mm. Patients were divided into two groups based on whether their calculated personalized margin ( ${\text{M}}_{\text{trans}+\text{rot}}^{'}$ ) exceeded 2 mm. This grouping enabled exploration of the prognostic impact of margins smaller than the theoretically required value when rotational errors were considered. Additionally, referencing previously reported threshold values (21–24), subgroup analyses were conducted using of $\bar{d}$ 30 mm as the cutoff to investigate the effect of target-isocenter distance on tumor control under rotational errors within ±3°.

Primary endpoints were intracranial progression-free survival (iPFS) and local control (LC), with secondary endpoints including intracranial local failure (ILF) and overall survival (OS). iPFS was defined as the duration from FSRT initiation until intracranial lesion progression or the last follow-up. LC was defined as the absence of intracranial progression in irradiated regions throughout follow-up or until death. ILF was defined as lesion progression within irradiated regions during follow-up. OS was defined as the time from FSRT initiation to death or last follow-up if still alive. Disease progression was evaluated using the Response Evaluation Criteria in Solid Tumors version 1.1 criteria (25).

### 2.5 Statistical analysis

Analyses were performed using SPSS v26.0 and R v4.4.2. Continuous variables were reported as mean ± SD for normally distributed data, median and range for non-normally distributed data, and categorical variables were presented as frequencies and percentages. Comparisons utilized the t-test, Wilcoxon rank-sum test, or chi-square test as appropriate. Kaplan–Meier survival curves and log-rank tests compared iPFS and OS. Spearman correlation assessed variable relationships. Cox proportional hazards regression analyzed prognostic factors for iPFS and OS. Logistic regression explored relationships between target-isocenter distances, rotational errors, LC, and ILF. Multivariate linear regression assessed independent impacts of metastasis characteristics and treatment parameters on rotational errors and PTV margins. Statistical significance was defined as *P < 0.05*.

## 3 Results

### 3.1 Patient characteristics and plan statistics

**Figure 1:**
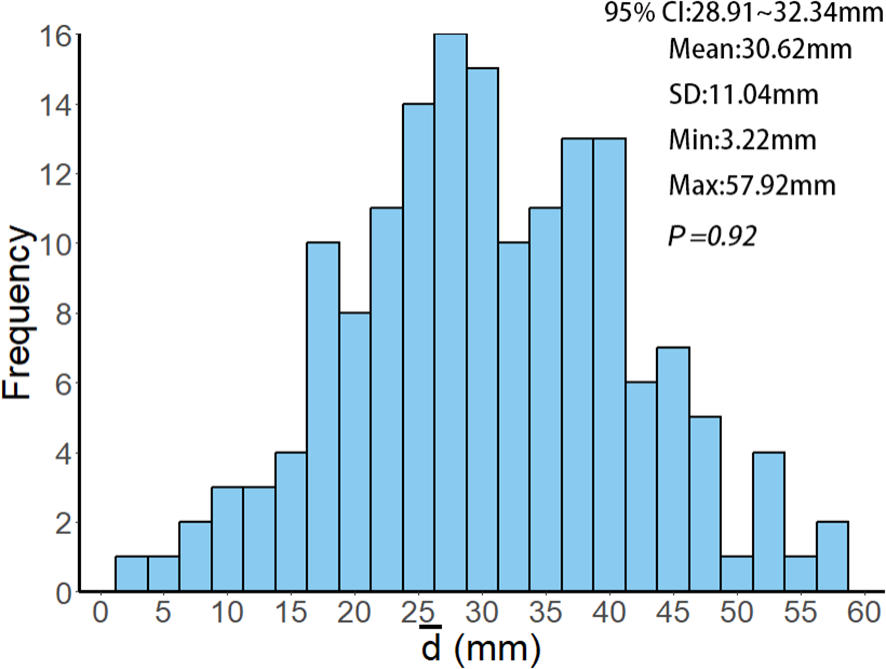
Histogram of the frequency distribution of $\bar{d}$ values among 161 patients. $\bar{d}$ represents the average distance of each patient's target volume geometric center to the treatment isocenter, calculated using Equations 1~2. 95% CI, SD, Min and Max represent the 95% confidence interval, standard deviation, minimum value and maximum value of the $\bar{d}$ values, respectively. *P = 0.92* indicates that the Kolmogorov-Smirnov test, used to analyze the $\bar{d}$ values of all patients, confirms the data follows a normal distribution (*P = 0.92 > 0.05*).

This retrospective study analyzed patients with multiple brain metastases treated by linear accelerator-based FSRT at Jiangsu Cancer Hospital from January 2021 to December 2023. A total of 161 patients with 391 lesions were included, undergoing 733 treatments. As detailed in **Table 1**, the mean age was 61.42 ± 9.57 years; 88 patients (54.66%) were male, and 73 (45.34%) were female. The median Karnofsky Performance Status (KPS) was 80 (range: 60~100). The primary tumor was predominantly lung cancer (75.78%, n=122), with a median of two brain metastases per patient (range: 2-8), a median maximum lesion diameter of 16.1 mm (range: 3~70.6 mm), and a median lesion volume of 2.98 cm³ (range: 0.11~188.24 cm³). Patients underwent a median of 3 fractions (range: 2~15). Regarding treatment planning, the median Conformity Index (CI) was 1.03 (range: 0.92~1.89), mean Homogeneity Index (HI) was 0.13 ± 0.06, median D_2%_ (the minimum dose delivered to the hottest 2% of the volume) was 34.78 Gy (range: 20.45~68.66 Gy), median D_50%_ (the minimum dose delivered to the hottest 50% of the volume) was 32.75 Gy (range: 20.29~64.09 Gy), and median D_98%_ (the minimum dose delivered to the hottest 98% of the volume) was 29.83 Gy (range: 19.94~59.45 Gy). The average distance from lesion geometric centers to the isocenter was 30.62 ± 11.04 mm (95% confidence interval (95% CI): 28.91~32.34 mm; **Figure 1**).

**Table 1:** Patient Baseline Characteristics.

Characteristic	Value
Gender, NO (%)	
Female	73 (45.34)
Male	88 (54.66)
Age, years, mean ± SD	61.42 ± 9.57
KPS, percentages, median (range)	80 (60~100)
Primary Site, NO (%)	
Esophagus	9 (5.59)
Lung	122 (75.78)
Breast	14 (8.70)
Other	16 (10)
Number of BM, NO, median (range)	2 (2~8)
Maximum Diameter of BM, mm, median (range)	16.1 (3.0~70.6)
Volume of BM, cm³, median (range)	2.98 (0.11~188.24)
CI, median (range)	1.03 (0.92~1.89)
HI, mean ± SD	0.13 ± 0.06
D_2%_, Gy, median (range)	34.78 (20.45~68.66)
D_50%_, Gy, median (range)	32.75 (20.29~64.09)
D_98%_, Gy, median (range)	29.83 (19.94~59.45)
Fractions, NO, median (range)	3 (2~15)
$\bar{d}$ , mm, mean ± SD	30.62 ± 11.04

NO, number; SD, standard deviation; KPS, karnofsky performance status; BM, brain metastases; CI, conformity index; HI, homogeneity index; Gy, gray. D_2%_ represents the minimum dose received by the hottest 2% of the target volume the dose. D_50%_ represents the minimum dose received by the hottest 50% of the target volume the dose. D_98%_ represents the minimum dose received by the hottest 98% of the target volume the dose. $\bar{d}$ represents the average distance from each patient's target volume geometric center to the treatment isocenter.

### 3.2 Geometric accuracy

**Figure 2:**
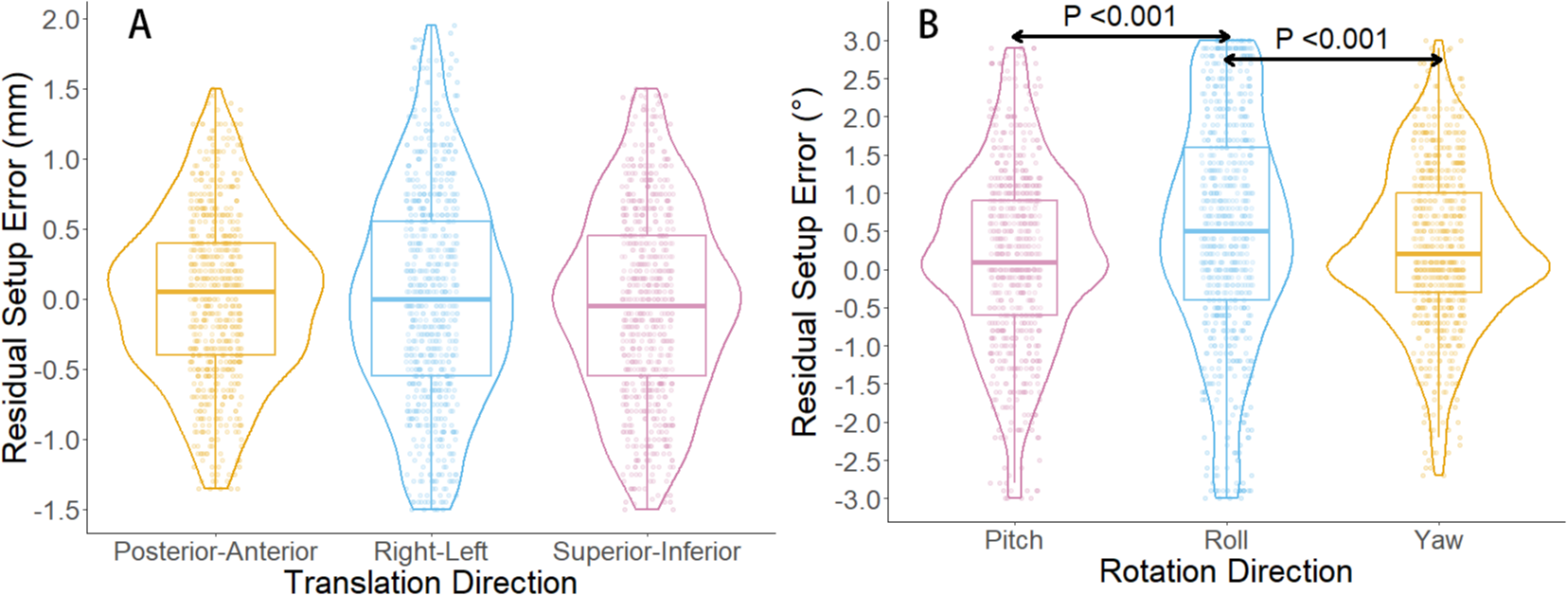
Boxplot and violin plot of residual setup errors in six directions. **(A)** shows the distribution of residual setup errors in three translational directions. **(B)** displays the distribution of residual setup errors in three rotational directions. In **(B)**, significant differences are observed in the residual setup errors between the roll direction and the yaw and pitch directions (*P < 0.001*, Wilcoxon rank-sum test).

**Figure 3:**
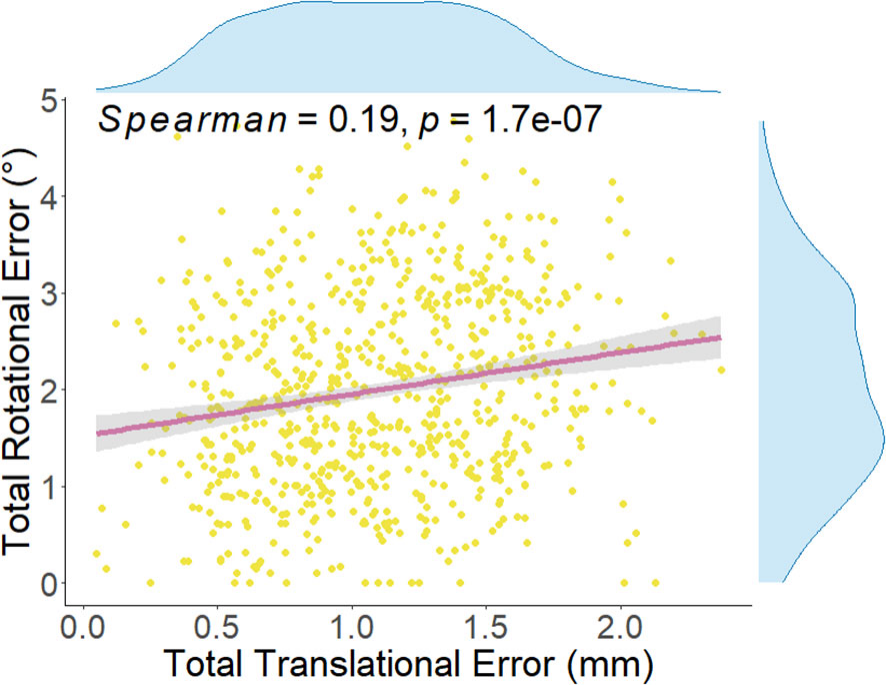
Spearman correlation analysis between total translational error and total rotation error for each treatment. The correlation coefficient (*Spearman = 0.19, P = 1.7e-0.7*) indicates a statistically significant relationship between the two. The total translational error for each treatment is calculated using Equation 4, while the total rotation error is calculated using Equations 5~8.

In this study, the six-dimensional residual setup error data for 733 treatments after KV-CBCT correction are presented in **Figure 2**. In the translational directions (**Figure 2A**), the average residual errors were as follows: posterior-anterior direction: 0.01± 0.59 mm; superior-inferior direction: – 0.04 ± 0.68 mm); right-left direction: –0.001 ± 0.75 mm). In the rotational directions (**Figure 2B**), the average residual errors were as follows: yaw: 0.30° ± 1.05°); pitch: 0.15° ± 1.17°); roll: 0.49° ± 1.48°). Inter-group comparisons for the three rotational directions (Wilcoxon rank-sum test) revealed significant differences between roll and both yaw and pitch (*P < 0.001*) (**Figure 2B**). Further analysis showed a significant positive correlation between ${\text{R}}_{\text{total}}$ and ${\text{T}}_{\text{total}}$ (*P < 0.01*), though the correlation coefficient (*r = 0.19*) suggests a weak association between the two (**Figure 3**).

Further analysis (**Table 2**) examined correlations between setup errors (rotational and translational) and lesion number, maximum diameter, cumulative volume, and fractions. Spearman correlation analyses revealed weak positive correlations between rotational errors and cumulative lesion volume (r = 0.20, *P = 0.01*) and maximum diameter (r = 0.24, *P < 0.01*), and between cumulative lesion volume and translational errors (r = 0.16, *P = 0.04*). Multivariate linear regression confirmed maximum lesion diameter as an independent predictor of rotational error (Beta = 0.24, 95% CI: 0.02~0.27, *P = 0.02*); other factors (fraction number, cumulative volume, lesion number) showed no significant independent effects (*P = 0.24, 0.96*, and *0.62*, respectively). No factors showed statistical significance in predicting translational errors (diameter, *P = 0.07*; fractions, *P = 0.12*; volume, *P = 0.59*; lesion number, *P = 0.28*).

**Table 2:** Spearman correlation analysis between tumor and treatment parameters versus setup errors and PTV margins.

Variables	${\bar{R}}_{total}$		${\bar{T}}_{total}$		${\text{M}}_{\text{trans}+\text{rot}}^{'}$	
	r	*P*	r	*P*	r	*P*
Number of BM	0.07	0.37	–0.05	0.53	–0.21	0.01
Volume of BM	0.20	0.01	0.16	0.04	–0.01	0.91
Maximum Diameter of BM	0.24	< 0.01	0.14	0.07	0.01	0.90
Fractions	–0.12	0.12	–0.12	0.12	0.25	< 0.01

${\bar{R}}_{total}$ denotes the average of ${\text{R}}_{\text{total}}$ across all treatment fractions per patient, where ${\text{R}}_{\text{total}}$ represents the overall rotational error per treatment calculated using Equations 5-8. ${\bar{T}}_{total}$ denotes the average of ${\text{T}}_{\text{total}}$ across all treatment fractions per patient, where ${\text{T}}_{\text{total}}$ represents the overall translational error per treatment calculated using Equation 4. ${\text{M}}_{\text{trans}+\text{rot}}^{'}$ indicates the individualized PTV margin per patient derived from the Jenghwa Chang model, as detailed in Equation 13. BM, brain metastases. R, correlation coefficient.

### 3.3 Margin calculation results

In this study, the random errors σ of the six-dimensional residual setup errors (including posterior-anterior, superior-inferior, right-left, yaw, pitch, and roll directions) for 161 patients were 0.54 mm, 0.54 mm, 0.64 mm, 0.79°, 0.80°, and 1.14°, respectively. The system errors Σ were 0.42 mm, 0.52 mm, 0.53 mm, 0.84°, 0.97°, and 1.15° (**Table 3**). Using Van Herk formula (Equations 9 **~** 11), the PTV margin expansion values for the three translational directions (posterior-anterior, superior-inferior, right-left) were calculated to be 1.44 mm, 1.68 mm, and 1.78 mm, respectively. Furthermore, the results (**Table 3**) show that the standard deviations of the ${\text{T}}_{\text{total}}$ and ${\text{R}}_{\text{total}}$ for all patients, after processing with KV-CBCT and the 6-DOF treatment couch, were 0.44 mm and 1.02°, respectively. When both translational and rotational factors are considered, the combined PTV margin expansion ${\text{M}}_{\text{trans}+\text{rot}}$ calculated using the Jenghwa Chang formula is 1.74 mm with $\bar{\text{d}}$ at its mean value (i.e., 30.62 mm, see Equation 3). When $\bar{\text{d}}$ is considered within the 95% CI (i.e., 28.91~32.34 mm), the corresponding ${\text{M}}_{\text{trans}+\text{rot}}$ values range from 1.69~1.79 mm (**Table 3**).

**Table 3:** Planning target volume margins calculation.

	Margin with Translational Errors						Margin with Translational & Rotational Errors	
	${\text{T}}_{\text{PA}}$ (mm)	${\text{T}}_{\text{IS}}$ (mm)	${\text{T}}_{\text{RL}}$ (mm)	${\text{R}}_{\text{Yaw}}$ (°)	${\text{R}}_{\text{Pitch}}$ (°)	${\text{R}}_{\text{Roll}}$ (°)		
Mean	0.01	–0.04	–0.001	0.30	0.15	0.49	${\text{σ}}_{{\text{T}}_{\text{total}}}$ (mm)	0.44
SD	0.59	0.68	0.75	1.05	1.17	1.48	95% CI for $\bar{\text{d}}$ (mm)	28.91~32.34
σ	0.54	0.54	0.64	0.79	0.80	1.14	${\text{σ}}_{{\text{R}}_{\text{total}}}\;$ (°)	1.02
Σ	0.42	0.52	0.53	0.84	0.97	1.15	${\text{χ}}_{\text{α}}$	2.795
${\text{M}}_{\text{Van Herk}}$ (mm)	1.44	1.68	1.78	/	/	/	${\text{M}}_{\text{trans}+\text{rot}}$ (mm)	1.69~1.79

The standard deviation of the total translational error ( ${\text{σ}}_{{\text{T}}_{\text{total}}}$ ) represents the standard deviation of the total translational error ( ${\text{T}}_{\text{total}}$ ) for all 733 treatments across the 161 patients ( ${\text{T}}_{\text{total}}$ is calculated using Equation 4). ${\text{T}}_{\text{PA}}$ , ${\text{T}}_{\text{IS}}$ , ${\text{T}}_{\text{RL}}$ , ${\text{R}}_{\text{Yaw}}$ , ${\text{R}}_{\text{Pitch}}$ , ${\text{R}}_{\text{Roll}}$ represent the residual setup errors in the posterior-anterior, superior-inferior, right-left, yaw, pitch, and roll directions, respectively. SD refers to the standard deviation. σ represents the group random error, calculated using Equation 11. Σ represents the group systematic error, calculated using Equation 10. ${\text{M}}_{\text{Van Herk}}$ represents the planning target volume (PTV) margin expansion calculated using Van Herk’s formula for the 161 patients, considering only the residual setup error in the translational directions. ${\text{T}}_{\text{total}}$ represents the total translational error for each patient before every treatment. ${\text{σ}}_{{\text{T}}_{\text{total}}}$ is the standard deviation of ${\text{T}}_{\text{total}}$ for all 733 treatments across 161 patients. “95% CI for $\bar{d}$ ” denotes the 95% confidence interval of $\bar{d}$ , which is the average distance from each patient's target volume geometric center to the treatment isocenter. ${\text{R}}_{\text{total}}$ represents the total rotational error for each patient before each treatment, calculated using formulas Equations 5~8, and ${\text{σ}}_{{\text{R}}_{\text{total}}}$ is the standard deviation of ${\text{R}}_{\text{total}}$ for all 733 treatments across the 161 patients. ${\text{χ}}_{\text{α}}$ represents the value corresponding to the probability of the gross tumor volume (GTV) being covered by the PTV. When the probability of GTV being inside the PTV is at least 95%, ${\text{χ}}_{\text{α}}$ is set to 2.795. ${\text{M}}_{\text{trans}+\text{rot}}$ represents the combined PTV margin expansion for the 161 patients, considering both rotational and setup errors, calculated using Jenghwa Chang formula.

Potential influencing factors for PTV margin were explored (**Table 2**). Spearman correlation analysis showed a weak positive correlation between fraction number and PTV margin (r = 0.25, *P < 0.01*) and a weak negative correlation with lesion number (r = –0.21, *P = 0.01*). Multivariate regression analysis, including lesion diameter, fraction number, cumulative volume, and lesion number, revealed no statistically significant independent predictors of PTV margin (diameter *P = 0.22*, fractions *P = 0.14*, volume *P = 0.28*, lesion number *P = 0.10*).

### 3.4 Prognostic outcomes

As of December 2024, the median follow-up was 16.87 months, with an ILF rate of 24.23% and a 1-year LC rate of 65.22%. Median iPFS was 12.87 months, median OS was not reached, with 1-year cumulative iPFS and OS at 50.92% and 70.80%, respectively.

Given the combined impact of rotational errors and target-isocenter distance on dose distribution, subgroup analyses were conducted based on a distance cutoff of 30 mm ( $\bar{\text{d}}$ ≤30 mm, n = 77; $\bar{\text{d}}$ >30 mm, n = 84). Results (**Table 4**) showed no significant differences in ILF rates (23.34% vs. 25.00%, *P = 0.81*) or 1-year local control rates (71.43% vs. 59.52%*, P = 0.11*). Kaplan-Meier analysis revealed no significant differences between groups in cumulative 1-year iPFS (50.62% vs. 49.97%, *P = 0.80*) or OS (66.18% vs. 73.81%, *P = 0.38*). Further analysis (**Table 5**) showed rotational errors did not significantly impact iPFS, OS, ILF, or local control rates (all *P > 0.05*).

**Table 4:** Analysis of the impact of distance from lesion to treatment isocenter on patient prognosis.

Endpoints	$\bar{\text{d}}\;\leq$ 30 mm Group (N = 77)	$\bar{\text{d}}\text{ >}$ 30 mm Group (N = 84)	*P*
one-year LC rates, %	71.43	59.52	0.11
ILF rates, %	23.34	25.00	0.81
1-year cumulative iPFS, %	50.62	49.97	0.80
1-year cumulative OS, %	66.18	73.81	0.38

$\bar{d}$ denotes the average distance from each patient’s lesion geometrical center to the treatment isocenter, calculated by Equation 2. LC, local control. ILF, intracranial local failure. iPFS, intracranial progression-free survival. OS, overall survival; N, number.

**Table 5:** Analysis of the impact of rotational errors on patient prognosis.

Endpoints	${\text{R}}_{\text{total}}$ ≤ 1° vs. 1°< ${\text{R}}_{\text{total}}$ ≤ 2°		${\text{R}}_{\text{total}}$ ≤ 1° vs. ${\text{R}}_{\text{total}}$ > 2°	
	HR/OR (95% CI)	*P*	HR/OR (95% CI)	*P*
one-year LC rates	1.82(0.35~9.40)	0.48	2.04(0.40~10.52)	0.39
ILF	1.25(0.24~6.52)	0.79	1.01(0.19~5.31)	0.99
iPFS	2.10(0.84~5.27)	0.12	1.93(0.77~4.84)	0.16
OS	1.65(0.51~5.36)	0.41	1.44(0.44~4.72)	0.55

The effects of rotational errors on iPFS and OS were evaluated using Cox proportional hazards regression models, while effects on intracranial local failure rate and 1-year local control rate were analyzed using logistic regression. ${\text{R}}_{\text{total}}$ denotes the overall rotational error per treatment calculated using Equations 5~8. LC, local control. ILF, intracranial local failure. iPFS, intracranial progression-free survival. OS: overall survival. HR, hazard ratio. OR, odds ratio; 95%; CI, 95% confidence interval.

Considering rotational errors, personalized margins ( ${\text{M}}_{\text{trans}+\text{rot}}^{'}$ ) were calculated (Equation 13), identifying 25 patients (15.53%) needing >2 mm margins. Comparing outcomes between patients requiring margins ≤2 mm vs. >2 mm revealed no statistically significant differences in 1-year cumulative iPFS (51.45% vs. 44.00%, *P = 0.85*), OS (69.84% vs. 71.78%, *P = 0.87*), ILF rates (22.79% vs. 32.00%, *P = 0.32*), or 1-year local control rates (65.44% vs. 64.00%, *P = 0.89*).

## 4 Discussion

In the current medical field, radiation therapy for multiple brain metastases has become a significant challenge in cancer treatment. In recent years, the application of SIMT non-coplanar VMAT technology for treating multiple brain metastases has been evaluated in numerous studies (10, 11, 23, 26). VMAT provides highly conformal dose distribution for multiple brain metastases by adjusting the intensity of beams, dose rates, and gantry rotation speed. Compared to coplanar VMAT, non-coplanar VMAT significantly improves the dose conformity and dose gradient of the PTV of brain metastases, especially when the lesions are close to each other (11). Furthermore, studies have shown that SIMT non-coplanar VMAT technology can reduce the total treatment time by nearly half while maintaining a high level of local control rate (10, 11). However, SIMT technology still faces several challenges in practical application, particularly with the introduction of rotational errors. As the distance from the target to the isocenter increases, the impact of rotational uncertainty on lesion dose coverage becomes more pronounced (5, 23, 26, 27). Roper et al. reported that when the distance between the isocenter and the target increases, a rotational error exceeding 1°can significantly lower the expected dose coverage of the target (5). Moreover, Sagawa et al. found that in multiple brain metastases, as the rotational error increased, the V_10Gy_ (the volume receiving at least 10 Gy) to V_16Gy_ (the volume receiving at least 16 Gy) values for the brain significantly increased, indicating that rotational errors may elevate the risk of tumor recurrence and normal brain tissue damage (27). These findings emphasize the necessity of controlling and correcting rotational errors when using SIMT technology to ensure the precision and safety of treatment.

Based on this background, the present study systematically analyzed residual six-dimensional setup errors. As shown in **Table 3**, average translational residual errors ranged from –0.04~0.01 mm, and rotational residual errors ranged from 0.15°~0.49°. According to the AAPM Task Group 142 report, residual setup errors after KV-CBCT correction and 6DOF couch adjustment should be within 1 mm and 0.5° (28). Recently, several studies have advocated even stricter criteria (14, 29). For instance, Carminucci et al. reported average residual translational errors of 1.67, 0.73, and 0.75 mm in posterior-anterior, superior-inferior, and right-left directions, respectively, and average rotational errors of 0.73°, 1.44°, and 0.76° in pitch, roll, and yaw directions during stereotactic radiotherapy (29). Compared to these studies, our results demonstrated superior setup accuracy for FSRT based on the SIMT non-coplanar VMAT technique, surpassing those commonly required in traditional whole brain radiotherapy (WBRT). Previous reports have indicated WBRT systematic translational errors ranging from –0.63 mm to 0.73 mm, with random errors of 0.75~1.39 mm (30). Such comparisons underscore FSRT’s distinct suitability for treating multiple brain metastases in the era of precision radiotherapy. Notably, the present study found significantly larger setup errors in the roll direction compared to pitch and yaw (**Figure 2B**). This observation aligns with previous literature and clinical experience, reflecting the greater practical difficulty in controlling roll rotations (31). Furthermore, we observed a statistically significant positive correlation between ${\text{R}}_{\text{total}}$ and ${\text{T}}_{\text{total}}$ (r = 0.19, *P < 0.01*; **Figure 3**). Although the correlation is relatively weak, it suggests an upward trend in translational errors with increasing rotational deviations. This finding corroborates earlier research (32, 33); Keeling et al. reported a close relationship between translational uncertainty and couch rotation angles in stereotactic radiotherapy (32).

The mechanisms underlying setup errors are complex, involving multiple contributing factors. Previous studies have identified several elements that may exacerbate these errors. Firstly, during the image guidance and registration process for multiple brain metastases, the relative motion between lesions can make it difficult for the 6-DOF treatment couch with CBCT to effectively correct all positioning uncertainties (6–8). Secondly, image registration and correction may lead to extended treatment times, which could increase the uncertainty of intrafraction motion (26, 34). Tarnavski et al. found that when the treatment duration exceeds 10 minutes, the probability of patient movement greater than 2 mm or 2 ° significantly increases (34). Furthermore, Schmidhalter et al. pointed out that, in non-frame fixation systems, some positioning inaccuracies may be related to weight loss in patients during radiotherapy, leading to increased movement space within the mask (35). This study did not analyze the impact of intrafraction motion. Although previous studies have suggested using CBCT before and after treatment to reduce the impact of setup errors, this measure was not implemented in our center due to economic and time constraints (36). To further elucidate potential causes of setup errors, we analyzed the influence of factors such as lesion count, maximum diameter, cumulative tumor volume, and number of treatment fractions (**Table 2**). The results indicated that cumulative tumor volume and maximum lesion diameter showed a weak but significant positive correlation with setup errors, particularly rotational errors, with lesion diameter demonstrating a significant independent association (*P = 0.02*). This suggests that larger lesions are more prone to increased setup and registration errors. A plausible explanation is that tumors undergoing radiation-induced necrosis or volume reduction may shift their relative position with respect to the skull, thereby affecting the accuracy of target registration, especially when employing bone-based alignment methods. This phenomenon is particularly pronounced with larger tumors or larger cumulative volumes (37). In summary, although overall setup errors in our study were within an acceptable range, a trend toward increased errors was observed in patients with larger lesions. Clinically, this highlights the necessity of more stringent monitoring and correction of setup errors for such patients, to ensure precise radiation dose delivery.

For FSRT based on SIMT and non-coplanar VMAT frame-free fixation techniques, some studies strongly recommend that radiation therapy centers define dedicated margins around metastatic lesions to minimize the negative impact of setup errors (38). Many studies, based on different considerations, have proposed various formulas for calculating PTV margin expansion (12, 14–18). Among these, the formula proposed by Van Herk is widely used for calculating PTV margin expansion based on translational errors (12). This formula aims to ensure that at least 90% of patients receive 95% of the prescribed dose coverage and has become a standard practice in clinical settings. In this study, the random error σ for the translational directions of 161 patients across 733 treatments ranged from 0.54~0.64 mm, with system errors Σ ranging from 0.42~0.53 mm (**Table 3**). Using the Van Herk formula, the PTV margin expansions for the posterior-anterior, superior-inferior, and right-left directions were 1.44 mm, 1.68 mm, and 1.78 mm, respectively, which aligns with results from previous studies (7, 39, 40). However, because the Van Herk formula does not fully account for rotational errors, it may underestimate the actual required PTV margin expansion in the application of SIMT technology (12). To overcome the limitations of the Van Herk formula, Jenghwa Chang proposed a statistical model-based method that integrates both translational and rotational errors to derive a PTV margin expansion formula that ensures the probability of the GTV being within the PTV region exceeds the predefined coverage probability (15, 16, 19). This formula provides a more accurate and universally applicable calculation for the expansion margin by considering factors such as the distance from the target volume to the isocenter, the confidence corresponding to the probability of GTV being covered by the PTV, and the six-dimensional setup errors, thereby improving upon the Van Herk formula (15, 16, 19). In this study, when $\bar{\text{d}}$ lies within its 95% CI (28.91~32.34 mm), the PTV margin expansion determined by Jenghwa Chang formula ranges from 1.69~1.79 mm. It is noteworthy that when $\bar{d}$ is taken as the average distance between all targets and the treatment isocenter for all patients (i.e., the D value, see Equation 3), according to the results in **Table 3**, with ${\text{σ}}_{{\text{T}}_{\text{total}}}=0.44\text{ mm}$ and a required GTV coverage probability of 95%, the initial translational margin ${\text{M}}_{\text{trans}}$ without considering rotation errors is 1.23 mm (i.e., $M_{trans}=\sqrt{{\left(\chi_{\alpha }\times \sigma_{T_{total}}\right)}^{2}}=\sqrt{{\left(2.795\times 0.44\right)}^{2}}=1.23\;mm$ ). If rotation errors are not considered, the GTV coverage probability drops to 73% when the rotational uncertainty $\sigma_{r_{total}}$ is 0.44 mm (i.e., $\sigma_{r_{total}}=0.01424\times D\times \sigma_{R_{total}}=0.44\;mm$ ), as $\chi_{\alpha }^{,}=\frac{M_{trans}}{\sqrt{(\sigma_{T_{total}}^{2}+\sigma_{r_{total}}^{2}})}=\frac{1.23}{\sqrt{(0.44^{2}+0.44^{2}})}=1.977$ , which corresponds to approximately 73% GTV coverage probability according to Jenghwa Chang’s study (15). When considering the rotational error ( $\sigma_{r_{total}}=0.44\;mm$ ), an additional 0.51 mm error compensation (i.e., $\Delta M=\chi_{\alpha }\sqrt{\sigma_{T_{total}}^{2}+\sigma_{r_{total}}^{2}})-M_{trans}=2.795\sqrt{(0.44^{2}+0.44^{2}})-1.23\;=\;0.51\;mm$ ) can prevent a reduction in the GTV coverage probability. The study revealed that when rotational errors are considered, the combined margin expansions, the combined margin ${\text{M}}_{\text{trans}+\text{rot}}$ ranging from 1.69 mm to 1.79 mm encompass most of the recommended values derived from the Van Herk formula, particularly aligning closely with its maximum recommended value of 1.78 mm in the right-left direction (**Table 3**). From a clinical standpoint, adopting a single, unified margin expansion is more practical and can improve consistency in both treatment planning and quality assurance. Therefore, taking both translational and rotational errors into account, this study proposes using the 95% CI’s upper bound derived from the Jenghwa Chang formula (1.79 mm) as the minimum safety reference for PTV margins in FSRT for multiple brain metastases at our center, thereby accommodating most patient setup errors and ensuring sufficient tumor dose coverage. It is noteworthy that the recommended PTV margin of 1.79 mm in our study contrasts significantly with the larger margins commonly used in WBRT, typically around 5 mm (41, 42). WBRT employs larger margins to ensure coverage of all potential metastatic sites, inevitably increasing radiation exposure to normal brain tissue and potentially causing long-term cognitive impairments (43). In contrast, our proposed FSRT margins substantially reduce the radiation dose to healthy brain tissue, simultaneously enhancing local control through precise dose delivery. This clearly underscores the clinical value and unique advantages of SIMT-based FSRT in treating multiple brain metastases.

Jenghwa Chang’s study points out that: 1. When the distance from the isocenter is small, the required compensatory margin for rotation errors increases slowly as the rotation error grows; however, when the isocenter distance significantly increases, the impact of rotation errors on dose deviation shows an “amplification effect,” leading to a steeper increase in the required margin expansion (19). 2. When the margin required to compensate for translational errors ( ${\text{M}}_{\text{trans}}$ ) is small, the impact of rotation errors and isocenter distance on the combined margin expansion ( ${\text{M}}_{\text{trans}+\text{rot}}$ ) becomes more significant (14). Taken together, these findings indicate the necessity and complexity of integrating both rotation uncertainty and isocenter distance into the determination of appropriate PTV margins for multiple brain metastases. Several other studies have also explored this nonlinear relationship between isocenter distance and rotation errors leading to off-target effects. For instance, Calmels et al. demonstrated that for very small targets (PTV margin: 2 mm, volume < 1 cm³), a rotational error of 1° at distances beyond 3 cm from the isocenter caused up to a 2.0% median decrease in GTV D_95%_ (21). Prentou et al. further proposed that, with a 1° rotational error, the distance from target to isocenter should be strictly limited to within 4 cm to prevent clinically relevant deterioration (>5%) in coverage, CI, and D_95%_ (22). Tsui et al. recommended restricting the isocenter distance to within 3.6~3.7 cm for rotational errors up to ±2° to maintain stable dose distribution (23). Additionally, Nakano et al. developed a geometric coverage loss model indicating that to limit geometric coverage loss due to rotational errors within 5%, lesions located beyond 7.6 cm from the isocenter should not be treated using SIMT techniques (24). They also emphasized an interaction effect between lesion size and isocenter distance, recommending a maximum isocenter-to-PTV distance of 5.5 cm for 1.5-mm lesions with a rotation error of 0.5°, and an even stricter limitation (3 cm) for lesions smaller than 2 cm³. Our study’s median lesion volume was 2.98 cm³, with rotation errors within ±3.0° and a mean distance from the lesion center to the isocenter of 30.62 mm, closely aligning with the recommended 3~4 cm threshold from the aforementioned studies (21–23). Collectively, these findings indicate that our study’s current isocenter distance effectively balances geometric precision and dose coverage requirements. Furthermore, caution should be exercised for lesions closely spaced (e.g., edge-to-edge distance <3 cm), as this might lead to overlapping dose hotspots (44). Given these considerations, we conducted subgroup analyses using 30 mm as a distance threshold. The results showed a consistent trend toward inferior clinical outcomes in tumor control for the subgroup with a distance greater than 30 mm ( $\bar{\text{d}}$ >30 mm) (see **Table 4**). Although the statistical significance between isocenter distance and clinical prognosis was not achieved, the observed dose-outcome trends corresponded well with the dose degradation patterns described by Calmels and Nakano et al. (21, 24). These observations suggest that with SIMT, when rotational errors are below 3°, isocenter distances greater than 3 cm may enter a region of dose coverage degradation, thereby necessitating stricter error management beyond this range. Furthermore, we explored the relationship between rotational errors and clinical outcomes but did not find statistically significant results (**Table 5**). Possible explanations include: (1) Substantial patient heterogeneity possibly masking the potential impact of rotational errors and isocenter distance on prognosis, considering the multifactorial nature of outcomes in brain metastases influenced by lesion volume, prescribed dose, KPS scores, systemic therapies, and primary tumor histology (45, 46). (2) Our standardized use of 1~2 mm PTV margins was adequate for the majority of patients, as theoretical calculations based on the Jenghwa Chang formula indicated that only 15.53% of patients required margins greater than 2 mm. This likely provided sufficient dose coverage for most patients under current margin strategies, diminishing the potential prognostic influence of geometric errors.

To comprehensively evaluate potential factors influencing the PTV margin, we further explored associations between lesion number, maximum lesion diameter, cumulative lesion volume, and the number of treatment fractions with PTV margins. The analysis revealed a weak positive correlation between treatment fractions and PTV margins and a weak negative correlation between lesion number and PTV margins (**Table 2**). However, subsequent multivariate regression analysis did not confirm independent effects of these factors, suggesting that the determination of PTV margins might be influenced by a combination of multiple factors. As previously mentioned, the impact of isocenter distance and setup errors may have greater significance. Additionally, although several studies have demonstrated the influence of target size on margin expansion (5, 21, 23), our study did not find a statistically significant correlation between lesion size and ${\text{M}}_{\text{trans}+\text{rot}}^{'}$ (*P = 0.91*). We speculate that using the average isocenter distance across all targets for individualized PTV margin calculation might have masked individual variations in lesion size, thus weakening the statistical correlation between the two variables. To further investigate the clinical applicability of the Jenghwa Chang formula, we divided patients based on the individualized ${\text{M}}_{\text{trans}+\text{rot}}^{'}$ value, using a cutoff of 2 mm. Although the differences between the two groups did not reach statistical significance, the group with ${\text{M}}_{\text{trans}+\text{rot}}^{'}$ > 2 mm demonstrated a higher ILF rate (32.0% vs. 22.8%), lower 1-year cumulative iPFS rate (44.00% vs. 51.45%), and slightly lower 1-year LC rate (64.00% vs. 65.44%). Clinically, most radiation oncology centers typically adopt PTV margins ranging from 0~2 mm (13). A survey from the Japanese Radiation Oncology Study Group also indicated 2 mm as the most commonly used margin (47). However, our study identified 25 patients whose theoretical margins exceeded 2 mm when rotational errors were considered, yet the clinical margins remained at 1~2 mm, potentially indicating a risk of suboptimal long-term local control. Previous research suggests that excessively large margins can increase the risk of radiation necrosis (36, 48), while insufficient margins may inadequately compensate for rotational errors, compromising local dose coverage. Given the observed adverse trends in ILF rates, 1-year LC, and cumulative iPFS for the ${\text{M}}_{\text{trans}+\text{rot}}^{'}$ > 2 mm group, We hypothesize that for patients with significant rotational errors, continuing to use an expansion margin within 2 mm may leave a risk of target miss, potentially leading to a decline in local control rates during follow-up. Based on this, this study suggests that for some patients with high rotational errors or excessively large distances from the isocenter, the expansion margin should be slightly increased to ensure the stability of local tumor control while balancing the risk of radiation necrosis.

This study, being a retrospective analysis, still has some limitations. Firstly, the study primarily relies on residual error data corrected by KV-CBCT and does not comprehensively quantify the dynamic movement of patients within fractions, which may lead to an underestimation of the true error. Previous studies that conducted CBCT scans before and after treatment have shown that the average intra-fraction motion in the translational direction ranges from 0.07 mm to 0.1 mm, and in the rotational direction, the average intra-fraction motion is between 0.027 ° and 0.109 ° . This suggests that future research at our center could consider real-time imaging monitoring or multiple CBCT scans to obtain a more comprehensive error evaluation (31). Secondly, the Jenghwa Chang formula assumes that the target volume is nearly spherical and applies uniform margin expansions in all directions. However, in clinical practice, the shapes of multiple lesions and the distances from the target volumes to the isocenter vary, which may lead to differences in the sensitivity of different target volumes to rotational errors. Although some studies have explored non-uniform margin strategies based on different target shapes and distances, the challenge remains to implement personalized margin expansions within the treatment planning system (14, 18). Finally, the results of this study, which incorporate rotational error into margin analysis, are based on theoretical models and single-center data, lacking validation through multi-center or prospective trials. Therefore, the generalizability and applicability of the conclusions drawn from this study need to be further verified with a larger sample size and longer follow-up periods.

## 5 Conclusion

Through retrospective analysis of SIMT-based non-coplanar VMAT for patients with multiple brain metastases, this study demonstrated acceptable geometric accuracy with residual translational setup errors ranging from –0.04~0.01 mm and rotational errors between 0.15°~0.49°. This indicates that the technique ensured high precision and dose consistency within our center. According to the Van Herk formula, required margins for posterior-anterior, superior-inferior, and right-left directions were 1.44 mm, 1.68 mm, and 1.78 mm, respectively. However, when accounting for rotational errors using the Jenghwa Chang formula, the comprehensive margins based on the 95% CI for isocenter distances ranged between 1.69~1.79 mm. Using the average distance of all lesions to their respective isocenters (30.62 mm), the Jenghwa Chang formula calculated a translational-only margin of 1.23 mm, with rotational uncertainty $\sigma_{r_{total}}$ at 0.44 mm. Ignoring rotational errors decreased the target coverage probability from 95% to 73%. Further subgroup analysis indicated that while differences in ILF rates, 1-year LC, and cumulative iPFS between patients with ${\text{M}}_{\text{trans}+\text{rot}}^{'}$ > 2 mm and ≤ 2 mm were not statistically significant, the former demonstrated unfavorable trends (higher ILF, lower LC and iPFS). This suggests that the current margin range (1~2 mm) may be inadequate to maintain long-term local control in patients with significant rotational errors. Moreover, subgrouping by average isocenter distance (30 mm threshold) indicated that patients with lesions situated over 3 cm from the isocenter might be at increased risk of compromised dose coverage when rotational errors were within ±3.0°, potentially affecting tumor control. In summary, this study recommends a minimum safe margin of 1.79 mm for SIMT-based non-coplanar VMAT FSRT in treating multiple brain metastases, ensuring sufficient coverage in most clinical scenarios and providing a basis for future treatment optimization.

## Data Availability

The raw data supporting the conclusions of this article will be made available by the authors, without undue reservation.
